# Young Adults’ Engagement With a Self-Monitoring App for Vegetable Intake and the Impact of Social Media and Gamification: Feasibility Study

**DOI:** 10.2196/13324

**Published:** 2019-05-10

**Authors:** Monica Nour, Juliana Chen, Margaret Allman-Farinelli

**Affiliations:** 1 School of Life and Environmental Sciences Charles Perkins Centre The University of Sydney Camperdown Australia

**Keywords:** vegetables, young adults, mHealth, social media, experimental game

## Abstract

**Background:**

Social media and gamification have been used in digital interventions for improving nutrition behaviors of young adults, but few studies measure engagement.

**Objective:**

This feasibility study aimed to explore user engagement with a 4-week smartphone program for improving vegetable intake.

**Methods:**

A goal setting and self-monitoring app was developed for feasibility testing. We assessed if additional components of gaming and/or social media support increased engagement. A 2 × 2 factorial study design was used with participants randomly allocated to each group. Engagement with the app (usage) was captured via inbuilt software, which recorded total days of app usage (duration) and the frequency of logging vegetable intake. Uptake of the social media (Facebook) content was measured by tracking views, likes, and comments on posts.

**Results:**

Out of the 110 potential participants who completed the prescreening questionnaire online, 97 were eligible (mean age 24.8 [SD 3.4]). In total, 49% (47/97) of participants were retained at 4 weeks. Attrition within the first week was the highest among users of the gamified app without social support (Facebook; *P*<.001). Over the intervention period, 64% (62/97) of participants logged into their app, with vegetable intake recorded on average for 11 out of 28 days. The frequency of recording decreased each week (mean 4 [SD 2] days in week 1 versus mean 2 [SD 2] days in week 4). No effects of gaming or social support on the frequency of recording vegetables or the duration of app engagement were found. However, regardless of the app type, the duration of app engagement was significantly associated with vegetable intake post intervention (*P*<.001). In total, 60% of Facebook posts were viewed by participants but engagement was limited to likes, with no comments or peer-to-peer interaction observed.

**Conclusions:**

As duration of usage was associated with vegetable intake, a deeper understanding of factors influencing engagement is needed. Dimensions such as personal attributes and the setting and context require further exploration in addition to content and delivery.

## Introduction

An association between increased vegetable consumption, reduced all-cause mortality, and death from cardiovascular disease and some cancers has been reported [1]. Poor vegetable consumption is a global concern, with the World Health Organization launching a joint initiative with the Food and Agriculture Organization to improve intake [2]. Population-wide consumption of vegetables is inadequate [3,4], but young adults are the lowest consumers among adults in Australia [3] and the United States [4]. The Australian Dietary Guidelines recommend 5 and 6 serves of vegetables daily for females and males, respectively, [5] but only 4.2% and 1.7% of young adult females and males, respectively, meet this requirement [6]. In addition to consuming inadequate amounts, the variety of vegetables eaten is also poor [7]. This pattern of suboptimal intake is also observed in the United States, with 6.7% of 18 to 30-year-olds meeting the recommendation of 2.5 cups of vegetables per day [8-10]. Clearly, interventions are needed to improve intake.

This age group is typically less risk aversive and unconcerned with longer term benefits of healthful behaviors [11-13]. Thus, novel age-relevant strategies are indicated to motivate behavior change [14,15]. Information and communication technology (electronic health) interventions may be a suitable approach to engage young adults in behavior change [16]. In 2016, it was estimated that 95% and 92% of young adults owned a smartphone in Australia and the United States, respectively [17]. Furthermore, 2018 statistics show that young adults in Australia and the United States are the most frequent users of multiple social media platforms [18,19], with 91% of Americans aged 18 to 29 years using their devices for social networking [20].

In the last decade, social media has been rapidly adopted for health promotion, education, and behavior change, as it aligns with the social cognitive theory [21,22]. The social support, interactive sharing of content, peer influence, and empowerment (reinforcement and gratification) that social media offers may motivate users and enhance engagement with interventions [23-25]. However, social networking alone may be insufficient to change behavior, as recent reviews indicate that these platforms are usually a part of multicomponent interventions and independent effects cannot be discerned readily [24-30].

Gamification, whereby game strategies are used to motivate behavior change, is another strategy that can be delivered using technology and is becoming popular in public health interventions [31]. Gaming has been shown to have positive impacts on physical activity levels and nutrition knowledge [29]. However, research demonstrating its effectiveness in improving nutrition behavior of young adults is limited [29].

The effectiveness of programs using modern information and communication technology to deliver theory-based behavior change interventions is dependent on user engagement [32]. Engagement refers to the manner in which users interact with an intervention and the amount or dose received (measured as time of interaction with intervention components) [33]. Digital interventions that do not have human support may be particularly susceptible to high dropout and nonusage attrition, in which participants do not remain engaged with intervention technologies [34,35]. However, few studies have reported on user engagement with electronic interventions. The available evidence indicates that dropout rates are high in social media–based studies [27], but it is suggested that the use of interactive strategies, gaming, or competitions may increase retention [30,36,37].

Public health programs need to be evidence-based and piloted to ensure that the selected features are effective and adaptable to the population at large. Generally, feasibility studies are used to test program components and determine whether they should be trialed in a larger intervention to measure impact on behavior [38]. Currently, the evidence base concerning the feasibility of gaming and social networking strategies in nutrition interventions is lacking [27,29,30].

This study has described the feasibility testing of a 4-week program using apps and social media to deliver an intervention to improve vegetable intake designed using the COM-B (Capability, Opportunity, Motivation and Behavior) system. We assessed if gaming and/or social media support increases engagement and has any additive effect on improvements in vegetable intake in young adults.

## Methods

### Intervention Components

**Figure 1:**
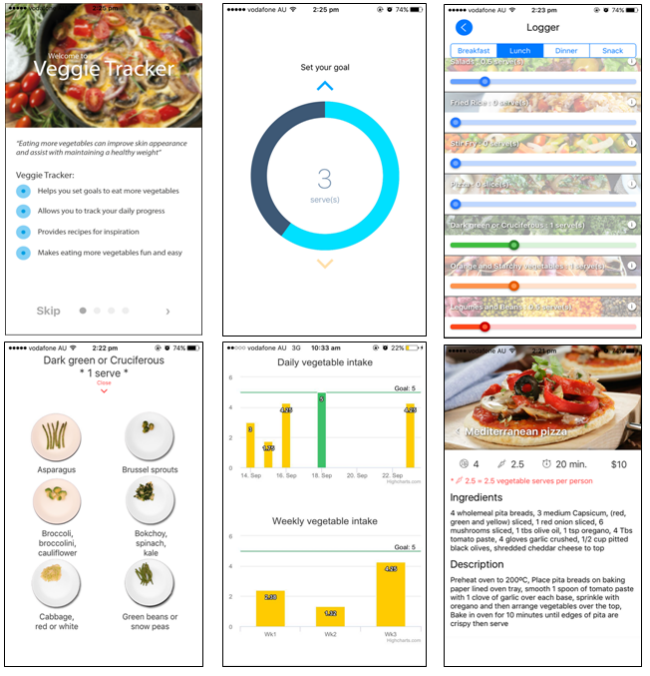
Screenshots of the standard self-monitoring app (left to right: welcome screen with instructions, goal setting screen, logging screen, information on serving sizes to assist with logging, progress screen, and recipe database).

**Figure 2:**
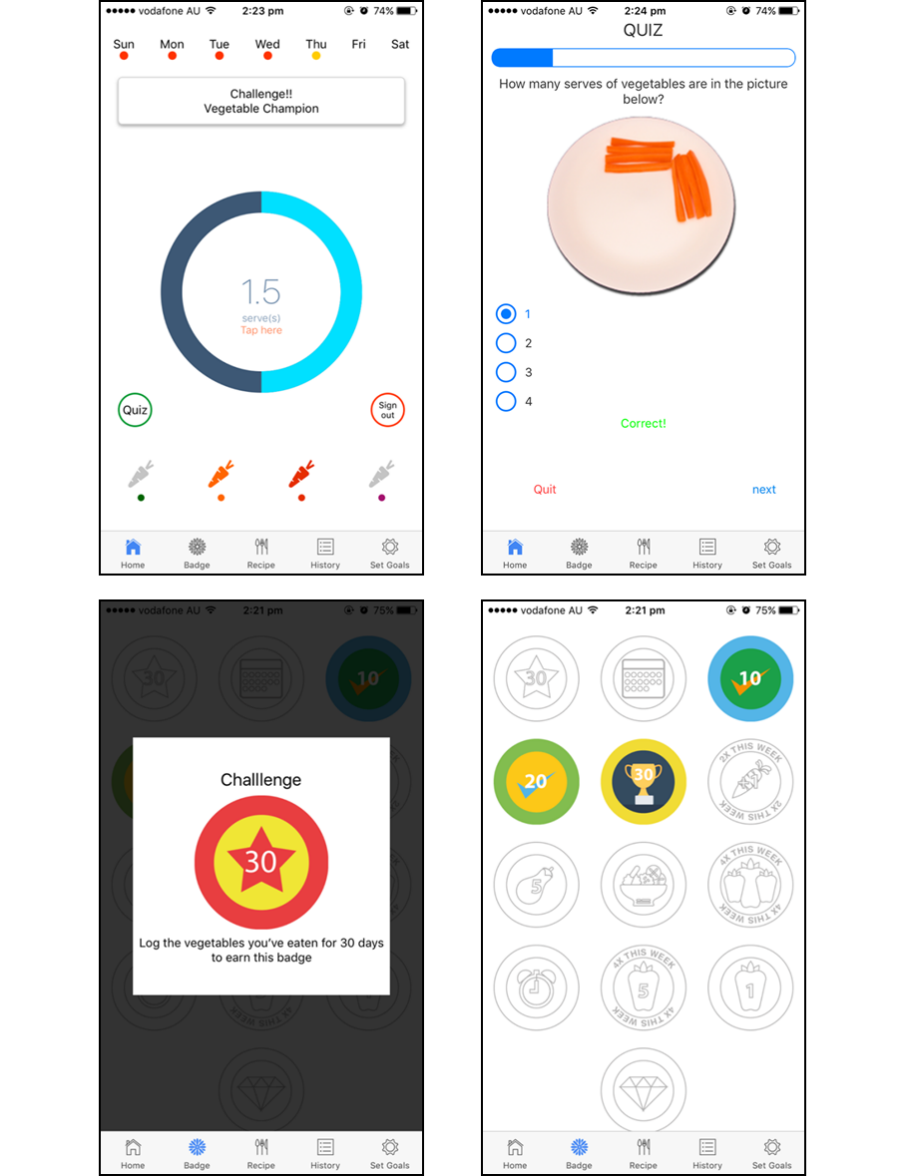
Screenshots of the gamified self-monitoring app (left to right: home screen, knowledge quiz, example challenge, badges to reward progress in knowledge, and other behaviors related to vegetable intake).

A goal setting and self-monitoring app with feedback on vegetable intake was developed by dietitians and experts in computer-human interaction. The app aimed to provide users the opportunity for self-evaluation of vegetable intake. The behavior change techniques (BCTs) of goal setting, self-monitoring, and the provision of feedback are successful strategies to maintain motivation and improve self-efficacy for practicing healthful lifestyle behaviors [14,39-42]. A second gamified version of the app was developed and incorporated rewards as incentivization. Figure 1 shows the standard self-monitoring app prototype, which featured the goal setting and recording components and a recipe database that was searchable by meal or ingredient. Figure 2 shows the gamified app prototype that had additional features, including challenges and badges as rewards for goal attainment. Providing rewards is recognized as an effective BCT for enhancing self-regulation by reinforcing the desired behavior [43,44]. The challenges were designed to be tailored to the user’s intake and were provided on a weekly basis. For example, if a user reports only consuming vegetables at dinner, they will be prompted with a *meal time challenge*, encouraging vegetables to be included in other meals such as breakfast, lunch, or snacks.

Additional program materials such as infographics, meal plans, and cooking videos were developed by a dietitian to address the key barriers to vegetable intake among young adults, including their poor knowledge of daily recommendations and serving sizes [45,46] and low confidence in planning meals that include vegetables and inadequate cooking skills [46,47]. These materials were designed for delivery within a private social support (Facebook) group. Facebook serves as both a platform for sharing additional resources and for providing participants with additional social support from the dietitian and other peers within the group. The provision of social support has been recognized as an effective technique for behavior change as it provides empowerment and positive peer pressure [48].

Tables 1-3 provide a summary of the selected intervention functions to support change in vegetable intake and suggested BCTs [49]. All program materials and the smartphone app prototypes were tested for acceptability and relevance in focus groups with a sample of the target audience. A structured focus group setting was used to present participants with mock Facebook posts to be evaluated. The young adults were also asked for feedback on app *wire frames*, that is, still images of the app pages, to determine the appropriate features to be included. A detailed explanation of the views and preferences of the young adults have been reported elsewhere [46]. The final app (usable product) was tested for functionality internally within the research team.

**Table 1:** A summary of the behavior change techniques selected, their context within the Capability, Opportunity, Motivation and Behavior framework, and their application within the platform.

Capability, Opportunity, Motivation and Behavior Framework			Description of behavioral determinant	Behavior change technique^a^	Application within the program		
					Standard app	Gamified app	Social support (Facebook) group
**Capability**							
	**Psychological**						
		Knowledge; Intervention function: Education	Knowledge of recommended vegetable intake and serve sizes; Understanding health benefits	Information about health consequences	Infographic on recommended daily intake and what constitutes a vegetable serve	Infographic (see Multimedia Appendix 1) on recommended daily intake and what constitutes a vegetable serve and quiz-based game on recommended vegetable intake and serve sizes according to Dietary Guidelines	Infographic (see Multimedia Appendix 1) on recommended daily intake and what constitutes a vegetable serve and Facebook posts on the health benefits of vegetables
		Self-monitoring and Feedback on behavior; Intervention function: Enablement and Education	Recording vegetable consumption and review discrepancy between current intake and goal to encourage continued improvement	Self-monitoring of behavior; Feedback on behavior	App enables user to enter serves of vegetables consumed at each meal and review progress against personalized goal	App enables user to enter serves of vegetables consumed at each meal and review progress against personalized goal	—^b^
	**Physical**						
		Skill building; Intervention function: Training	Cooking skills: practicing the process of cooking with vegetables	Demonstration of the behavior; Instruction on how to perform a behavior	Recipe database searchable by ingredient or meal type	Recipe database searchable by ingredient or meal type	Short cooking videos to model cooking with vegetables, with challenges to encourage young adults to practice cooking skills and upload pictures of their dish
**Opportunity**							
	**Physical**						
		Reducing barriers; Intervention function: Environmental restructuring	Addressing flavor, time, and cost as a barrier to vegetable intake by developing meal planning and budgeting skills	Prompts/cues	—	—	Facebook posts providing cues on how to enhance flavor of vegetables and plan meals on a budget
		Habit formation; Intervention function: Training	Prompt rehearsal and repetition of vegetable consumption	Habit formation	The app requires participants to monitor vegetable intake against their goal daily	The app requires participants to monitor vegetable intake against their goal daily	—

^a^Based on Susan Michie’s Behavior Change Taxonomy behavior change techniques [40].

^b^Not applicable.

**Table 2:** A summary of the behavior change techniques selected, their context within the Capability, Opportunity, Motivation and Behavior framework, and their application within the platform (continued)*.*

Capability, Opportunity, Motivation and Behavior Framework			Description of behavioral determinant	Behavior change technique^a^	Application within the program		
					Standard app	Gamified app	Social support (Facebook) group
**Opportunity**							
	**Physical**						
		Cues to action; Intervention function: Environmental restructuring	Providing reminders to consume vegetables to increase the likelihood of practicing the behavior; Creating healthy triggers within the environment to support increased vegetable consumption	Prompts/cues	—^b^	—	Providing tips on the Facebook page on how to maximize exposure to vegetables as a means of increasing consumption such as “Take your forgotten veg out of that bottom fridge draw and place on the top shelf so you’re reminded to cook with them”
		Cues to action; Intervention function: Enablement	Providing weekly challenges to increase vegetable intake	Graded tasks	—	Challenge based on personal opportunities, for example, add veg to breakfast	—
	**Social**						
		Social support; Intervention function: Enablement	Instigating positive peer rivalry to encourage vegetable intake	Social support (practical)	—	—	Competitions such as *best cooked vegetable dish*, *quirkiest vegetable of the week* between peers on the Facebook page
**Motivation**							
	**Reflective**						
		Cognitive strategies; Intervention function: Persuasion	Restructuring beliefs and perceived barriers by *debunking* myths about vegetables, for example, bad taste	Framing/reframing	—	—	*Myth busting* articles encouraging participants to reevaluate beliefs, for example, Top 5 ways to enjoy the taste of vegetables
	**Automatic**						
		Goal setting and self-monitoring; Intervention function: Enablement	Setting SMART^c^ goals for increasing vegetable intake	Goal setting (behavior); Review behavior goal(s)	App prompts user to set goal for vegetables serves/day, personalized based on current intake so it is achievable. Can assess progress against goal and recommended intake in review page	App prompts user to set goal for vegetables serves/day, personalized based on current intake so it is achievable. Can assess progress against goal and recommended intake in review page	—

^a^Based on Susan Michie’s Behavior Change Taxonomy behavior change techniques [40].

^c^SMART: Specific, Measurable, Achievable, Relevant, Time-bound.

^b^Not applicable.

**Table 3:** A summary of the behavior change techniques selected, their context within the Capability, Opportunity, Motivation and Behavior framework, and their application within the platform (continued)*.*

Capability, Opportunity, Motivation and Behavior Framework		Description of behavioral determinant	Behavior change technique^a^	Application within the program		
				Standard app	Gamified app	Social support (Facebook) group
**Motivation**						
	Rewards/Incentives; Intervention function: Incentivization	Increasing the value of consuming vegetables through rewards	Incentive	—^b^	Rewards (badges) provided for recording intake, consuming a variety of vegetables, achieving challenges and playing knowledge quiz	Competitions such as uploading a picture of a dish containing vegetables rewarded with a voucher
	Self-efficacy; Intervention function: Enablement and Modeling	Providing the opportunity to gain confidence in eating more vegetables by breaking the behavior up into small achievable tasks	Goal setting (behavior); Demonstration of the behavior	Weekly goals for increasing vegetable intake slowly	Weekly goals for increasing vegetable intake slowly. Weekly challenges providing easy ways to increase vegetables in the diet, for example, add some vegetables to breakfast	Cooking videos, meal planning resources and recipes, tips on simple ways to eat more vegetables, for example, “What’s for dinner tonight? Add in veggies, make a side salad or stir fried veg”
	Social support; Intervention function: Persuasion	Validating and reinforcing improvements in vegetable intake to encourage repetition of the desired behavior	Information about others’ approval	—	—	Participants can post pictures of vegetable dishes to receive positive reinforcement from the researchers

^a^Based on Susan Michie’s Behavior Change Taxonomy behavior change techniques [40].

^b^Not applicable.

### Study Design

Feasibility testing of the proposed program components was conducted using a 2 × 2 factorial study design (standard goal setting and self-monitoring app vs gamified self-monitoring app × social support vs no social support) with random allocation to each group. This design allowed for the comparison of 4 different conditions to determine which generated the best engagement. Participants were randomized into 1 of 4 groups. Each group was given access to a smartphone app for goal setting, self-monitoring, and the provision of feedback. In total, 2 groups received the additional gaming features within their app for incentivization and 2 groups (one using the gaming app and one using the standard app) received daily support and additional materials through a social support (Facebook) group. Multimedia Appendix 2 shows the 4 groups, their study conditions, and program components.

The reporting of outcomes was guided by the CONSORT E-HEALTH checklist of information to include when reporting on social media, serious games, or mobile health trials [50]. All study materials were delivered electronically. The study materials and methods were approved by the University Human Ethics Committee, approval number 2017/306.

### Participants and Recruitment

Young adults aged 18 to 30 years who owned a smartphone were eligible to participate. Pregnant women and those with a history of disordered eating or medical contraindications were excluded from this study. This study was advertised in the community at large throughout New South Wales (NSW) over 5 months between October 2017 and February 2018. Recruitment strategies included Facebook posts, flyers posted in tertiary education campuses and distributed through local club newsletters, and information stands. The recruitment flyers used the university logo to meet ethical requirements; however, affiliation with the institution did not appear on other program material. Recruitment advertisements were centered on eating more vegetables to improve health and well-being. For example, one Facebook advertisement stated “Feeling tired? Essential nutrients in vegetables can enhance your wellbeing. Learn how to eat a little more veg every day through the 4-week smartphone program designed by researchers at The University of Sydney.”

Participants expressing interest in this study were directed to a Web-based survey providing information about the study and eligibility screener. If eligible, participants were directed to the baseline questionnaire assessing usual vegetable intake and motivation for consuming vegetables. Informed consent was collected via this survey. Participants agreed that all data collected as part of the study could be shared within publications in a deidentified form. The survey was delivered using the Redcap software (Vanderbilt), a secure Web-based application for building and managing Web-based surveys [51]. As part of the consent, participants were informed that upon completing the 4-week intervention, they would be entered into a draw to win one of 4 Aus $25 grocery vouchers. Separate informed consent was obtained from 10 participants agreeing to participate in a postprogram interview. Respondents received a gift voucher valued at Aus $10 for participation in the interview.

### Randomization and Participant Instructions

Participants who met study eligibility and provided consent were enrolled into a study group by an independent researcher (JC). A Web-based number generator allowed randomization and stratification by gender [52]. The researcher collecting and analyzing the data was blinded to allocations throughout the duration of the study, data collection, and analysis (MN).

All participants were emailed an infographic educating on the recommended daily vegetable intake and what constitutes a vegetable serve (Multimedia Appendix 1) as well as a link to download the app for their allocated intervention group. The app was available at no cost from the Google Play or Apple store. They were instructed to set intake goals at baseline and use the app daily to record and monitor vegetable intake throughout the 4-week study period. Short pop-up instructions explained the functions of the app to users on first log in. The app was designed to reset the logging status at midnight each day. Throughout the 4-week intervention period, reminder text messages were sent to prompt recording of vegetable intake if a participant had not logged into the app for 3 consecutive days. Those randomized into a social media support group in addition to their designated app were invited via email to join the study social support (Facebook) group specific to their allocated intervention condition (gaming or nongaming). Participants were informed in the participant information sheet that the study comprised different *technologies* (ie, app and Facebook) and so could not be blinded completely. However, separate social support groups were created to avoid contamination between participants using the different apps (gaming vs nongaming). The dietitian used a predetermined schedule to share identical material within the social support (Facebook) groups each day for 4 weeks, with a total of 28 posts shared. Participants were asked to check the content posted daily. There was no human involvement other than the email correspondence for trial registration and regulation of Facebook posts. No counseling was given to participants.

After 4 weeks of using the designated app with or without the social media page, the participants received an email invitation to complete the follow-up questionnaire similar to baseline, but with additional questions asking for their experience/feedback on the program.

### Outcomes

The primary outcome of interest was the change in vegetable consumption (serves per day) at 4 weeks, including canned and frozen varieties but excluding fried potatoes. Engagement was measured as a secondary outcome.

### Data Collection

Demographic details, including age, gender, postcode (for categorizing socioeconomic status), education level, occupation, cultural background, and income were collected at baseline through a Web-based questionnaire. Vegetable intake (serves/day) was assessed at baseline and at the conclusion of the trial (4 weeks) using validated short questions [53], which quantified intake by the following vegetable groups: (1) potatoes (not fried), (2) salad vegetables, for example, lettuce/leafy vegetables, tomatoes, capsicum, and cucumber, (3) cooked vegetables, for example, zucchini, eggplant, sweet corn, green beans, and Asian greens, (4) legumes, for example, baked beans, lentils, and chickpeas, and (5) 100% vegetable juice. The questions asked participants to reflect on the last month and quantify the average servings they ate per day of each of the vegetable groups. Vegetable intake was reported using 9 response options (None, Less than 1 serving per day, 1 serving per day, 2 servings per day, 3 servings per day, 4 servings per day, 5 servings per day, 6 servings per day, and 7 or more servings per day). Autonomous and controlled motivation for consumption of vegetables was quantified using 4-point scale questions adapted from the self-regulation questionnaire [54,55], with a maximum possible score of 16. A higher score indicated greater motivation.

Engagement with the program was measured through usage (the uptake of intervention material) [56] and qualitative methods. Data related to the participant’s app usage were captured via an inbuilt software, which gives the time and date of app activity. The recorded log-ins were used as the measure of total days of app engagement (duration) and the data on recording of vegetable intake was used as an indicator of frequency of self-monitoring. Engagement with material posted within the social support groups was measured through user reactions such as *likes* and *comments* according to the Facebook definition of engagement [57]. This outcome measure reflects the ability of content to capture user attention rather than an estimation of total uptake. Uptake was measured using the *seen* data from Facebook analytics, which provides a list of people who have viewed the post.

Acceptability of program components was captured using a short postintervention questionnaire (all participants). The questionnaire included 4 5-point Likert scale questions as follows: “Rate how easy it was to use this program”; “Rate how much you liked this program”; “On a scale of 1-5, how likely would you recommend this program to others?”; and “How useful was the program to you?”

Qualitative assessment included semistructured interviews conducted via telephone. At conclusion of the 4-week trial, a random selection of participants received an email invitation to participate in a 15-min semistructured telephone interview for gathering subjective opinions of user experiences and feedback on the program components. The telephone interview was audio-recorded and transcribed for later thematic analysis.

### Data Analysis

Data analysis was conducted using IBM SPSS software for Windows version 22 [58]. Descriptive statistics were used to summarize the baseline characteristics of participants. Chi-squared tests were used to examine differences between groups in the education level, gender, and socioeconomic status. Analysis of variance was used to determine differences in baseline age and vegetable intake. Although the feasibility study numbers are not powered for detecting change, analysis of covariance was applied to measure the impact of the intervention on vegetable intake after 4 weeks (primary outcome) between groups, controlling for baseline vegetable intake. The analysis was by *intention to treat* with multiple imputation used for missing values so that all participants randomized at the commencement of the trial were retained for analysis regardless of compliance. In total, 5 imputed datasets were created based on age, gender, baseline vegetable intake, and Socioeconomic Index For Areas (SEIFA), which is a measure of the impact of the area of residence, rather than an individual’s income, occupation, or level of education, on intake. The imputed values were pooled using Rubin’s rule [59].

The differences in rates of attrition by group were assessed using chi-squared analysis for changes in proportions. Engagement with the app was explored quantitatively by summarizing log data by frequency of recording intake and period of app use by group. To investigate the relationship between the amount of app usage and changes in vegetable consumption, we used the Spearman correlation coefficient with significance assessed at *P*=.05. Facebook engagement was examined by totaling the *likes* and *comments*. Uptake of social media content was explored by calculating the percentage of participants who viewed each post. The most and least popular posts were determined using the following criteria—most popular: viewed by 80% or more of the study sample and least popular: viewed by 30% or less of the study sample. This criterion was based on previous literature, which shows that engagement can range from as low as 30% to as high as 73% [60,61]. Feedback collected through the follow-up semistructured interviews with participants was audio-recorded and later transcribed by one researcher who also coded into the NVivo Software program (QSR International Pty Ltd. Version 10, 2012). A thematic analysis using an open coding method and inductive approach was applied to group together common themes. Quotes were selected to represent the key themes.

## Results

### Participants

A total of 115 young adults expressed interest in participating in the study. Out of the 110 potential participants who completed the prescreening questionnaire online, 97 were eligible and randomized into one of the 4 groups. The breakdown of group allocation is displayed in Multimedia Appendix 2.

The characteristics and demographics of participants at baseline are presented in Table 4. The mean age was 24.8 (SD 3.4) years. The sample comprised 40% males. A total of 52 participants (54%) reported their highest level of education as a university degree or higher. The majority of participants (51/97, 53%) were of Australian or New Zealand descent, 1 of Aboriginal or Torres Strait Islander descent, 18 were of Asian descent, 5 of African descent, 18 of European descent, 1 of Middle Eastern descent, 1 of South American descent, and 2 of North American descent. The sample captured young adults across all socioeconomic areas, with 28% (n=27) categorized in the lower 2 SEIFA quartiles. There were no statistically significant differences found between the groups for: vegetable intake (*P*=.27), education (.79), gender (.95), and SEIFA (.3). The group using the standard app with social support (Facebook) was younger than the other 3 groups (*P*=.04). At baseline, the mean (SD) motivation score among all participants was 12.3 (1.8) out of a possible 16. A significant time effect was observed (*P*=.04) with an increase in motivation 4 weeks post intervention for all groups, but no group by time effect was found (*P*=.2).

### Attrition

In total, 10 participants withdrew from the intervention (10/97, 10%): 3 because of injury or illness; 4 because of lack of time; and 3 withdrew without giving a reason. At the end of the 4-week study period, participants were emailed the follow-up survey. Approximately half (47/97, 49%) completed this assessment (Multimedia Appendix 2). Females were more likely to complete the 4-week program than males (*P*<.001). The attrition rates by group and intervention week are presented in Table 5. The number of participants who dropped out after downloading the app in the first week of the intervention was significantly greater among the group who received the gamified app alone without social support (Facebook; *P*<.001). In the second week, drop out was highest for the group using the standard app with social support (Facebook; *P*<.001). Out of the 62 participants who downloaded the app, 29 (47%) remained engaged (logged vegetable intake) after 4 weeks. The overall proportion of completers was not significantly different between groups (*P*=.81).

**Table 4:** Baseline characteristics of participants by group condition.

Baseline characteristics		Standard app^a^	Gamified app^b^	Standard app with social support (Facebook)	Gamified app with social support (Facebook)	*P* value	
Age (years), mean (SD)		25.7 (3.2)^c^	24.6 (3.8)^d^	23.3 (3.0)^c,d^	25.5 (3.1)^c^	.04	
**Gender, n (%)**							
	Male	12 (44)	9 (41)	9 (38)	9 (38)	.95	
	Female	15 (56)	13 (59)	15 (62)	15 (62)	—^e^	
**Highest level of education, n (%)**							
	High school	7 (26)	5 (23)	7 (29)	4 (17)	.79	
	Diploma or certificate	7 (26)	5 (23)	6 (25)	4 (17)	—	
	University degree or higher	13 (48)	12 (54)	11 (46)	16 (66)	—	
**Socioeconomic Index For Areas, n (%)**							
	Quartile 1 (Lowest)	2 (7)	5 (23)	2 (8)	2 (8)	.3	
	Quartile 2	3 (11)	2 (9)	5 (21)	6 (25)	—	
	Quartile 3	10 (37)	3 (13)	3 (12)	4 (17)	—	
	Quartile 4	5 (19)	5 (2)	4 (17)	2 (8)	—	
	Quartile 5 (Highest)	7 (26)	7 (32)	10 (42)	10 (42)	—	
Vegetable intake (serves/day), mean (SD)		1.6 (1.4)	2.0 (1.5)	2.4 (1.3)	1.8 (1.6)	.27	

^a^Standard app for goal setting and self-monitoring with feedback.

^b^Gamified app for goal setting and self-monitoring with feedback with the addition of gamification. *P* values are for differences between groups using Tukeys post hoc analysis; shared subscripts represent statistically significant differences.

^c^*P*<.001.

^d^*P*=.003.

^e^Not applicable.

**Table 5:** The number of participants who dropped out of the program by group and by intervention week. Percentages are presented as proportions of those randomized.

Attrition by stage of intervention	Total, n (%)	Standard app, n (%)	Gamified app, n (%)	Standard app with social support (Facebook), n (%)	Gamified app with social support (Facebook), n (%)	*P* value for difference between groups ^a^
Randomized	97(100)	27 (28)	22 (22)	24 (25)	24 (25)	—^b^
Drop out week 1 (did not download app)	35 (36)	10 (37)	7 (32)	6 (25)	11 (46)	—
Drop out week 1 (after downloading app)	7 (7)	1 (4)	5 (23)	0 (0)	0 (0)	<.001
Drop out week 2	6 (6)	2 (7)	0 (0)	3 (13)	1 (4)	<.001
Drop out week 3	2 (2)	0 (0)	1 (4)	1 (4)	1 (4)	.005
Drop out week 4	0 (0)	0 (0)	0 (0)	1 (4)	0 (0)	.07
Completed 4 weeks	47 (49)	14 (52)	9 (41)	13 (54)	11 (46)	.81

^a^*P* value for differences in proportions between groups using chi-square tests.

^b^Not applicable.

### Change in Vegetable Intake

Significant differences in vegetable intake over time (*P*=.001) were found. However, no change was observed in the group by time differences (*P*=.43; Table 6).

### Engagement

#### Self-Monitoring Apps

**Figure 3:**
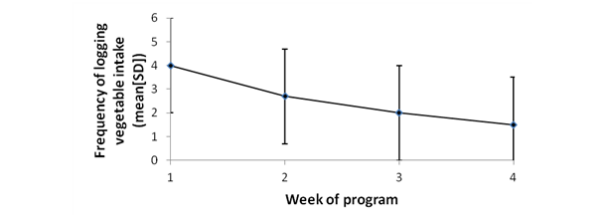
The mean frequency at which vegetable intake was recorded each week for the total sample (N=97), regardless of app type.

**Figure 4:**
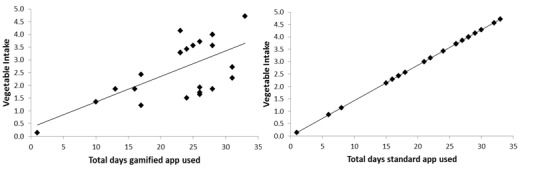
Correlation between total days of use of apps (with or without social support (Facebook)) and vegetable intake postintervention measured by validated short questionnaire. Gamified app: r=0.64; n=24; P=.001; Standard app: r=1; n=23; P<.00001.

**Figure 5:**
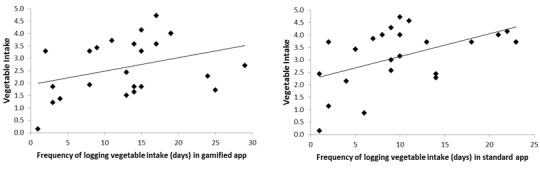
Correlation between frequency of logging (days) in the apps (with or without social support (Facebook)) and vegetable intake postintervention measured by a validated short questionnaire. Gamified app: r=0.35; n=24; P=.09; Standard app: r=0.49; n=23; P=.02.

Analysis of the app log data showed that 64% (62/97) of participants logged into the app. On average, each participant logged their vegetable intake on 11 out of 28 days during the intervention. The frequency of recording decreased each week for the overall sample (Figure 3). There were no significant differences between groups for frequency of recording intake and days of engagement with the app (Table 7). However, regardless of app type, the duration of app engagement (total days of use) was significantly positively associated with vegetable intake post intervention (*P*<.001; Figure 4). The frequency of recording was significantly associated with vegetable intake among those using the standard self-monitoring app only (Figure 5).

**Table 6:** Changes in vegetable intake from baseline to follow-up by group (N=97, using imputed dataset).

Group	Standard app^a^	Gamified app^b^	Standard app with social support (Facebook)	Gamified app with social support (Facebook)
Baseline, mean (SD)	1.6 (1.4)	2.0 (1.5)	2.4 (1.3)	1.8 (1.6)
Follow-up, mean (SD)	1.7 (1.2)	1.6 (1.2)	2.2 (1.6)	1.5 (1.2)
Change	0.1	−0.4	−0.1	−0.3
*P* value (time)	.001	—^c^	—	—
*P* value (group × time)	.43	—	—	—

^a^Standard app for goal setting and self-monitoring with feedback.

^b^Gamified app for goal setting and self-monitoring with feedback with the addition of gamification.

^c^Not applicable.

**Table 7:** Data on engagement with the app and Facebook material by group according to frequency of logging intake, days engaged with the app, uptake of Facebook material, and number of likes.

Group	Standard app	Gamified app	Standard app with social support (Facebook)	Gamified app with social support (Facebook)	*P* value for difference between groups^a^
Frequency of logging intake in app, mean (SD)	11 (7)	8 (5)	11 (7)	14 (8)	.30
Number of days engaged with app (log-ins), mean (SD)	23 (9)	20 (8)	22 (9)	23 (6)	.80
Uptake of Facebook material (posts seen), %	—^b^	—	58.4	61.2	.80
Engagement with Facebook material, number of likes	—	—	32	46	.30

^a^*P* value for differences between groups using analysis of variance.

^b^Not applicable.

#### Facebook Posts

##### Uptake (Views)

**Figure 6:**
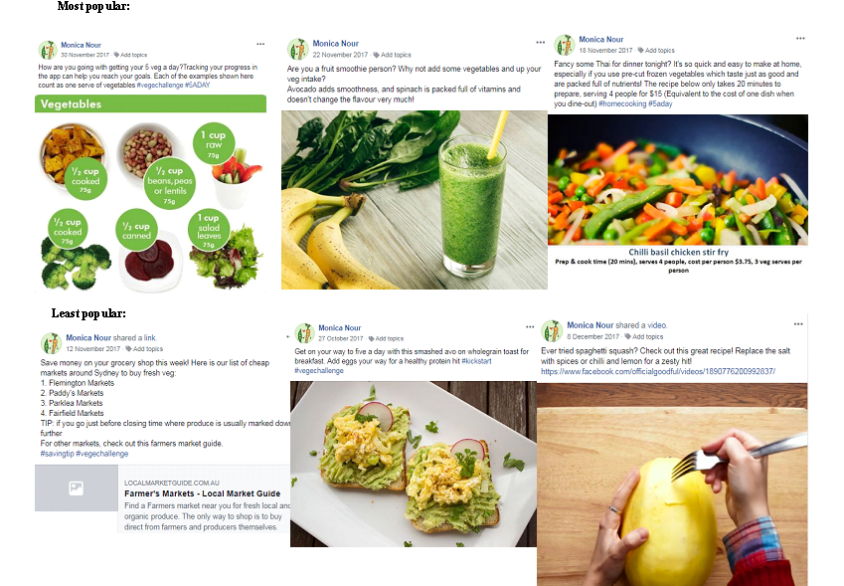
Examples of most (top row) and least popular (bottom row) posts.

Uptake of Facebook posts (percentage of posts viewed by participants) did not differ between the group that used the gaming app and the group that used the standard app (mean [SD] percentage of posts seen by participants; 61.2 [22.1] and 58.4 [23.9], respectively; Table 7). The percentage of posts viewed was well maintained over the intervention period. The mean percentage of views was 63.5 in week 1 (SD 17.8) and 62.5 in week 4 (SD 10.2). The most popular Facebook posts (ie, viewed by ≥80% of participants) were recipes with time-saving elements (eg, using frozen vegetables) and those that offered vegetable preparation hacks such as how to quickly chop a capsicum. Meal inspiration posts that suggested new ways to try vegetables such as by adding spinach to smoothies, making vegetable-based dips, or adding beans to salads were well received. Meal planning information was also very popular (particularly posts that featured a weekly meal plan and shopping list). Additionally, uptake was high on posts that suggested money saving tips and used infographics to pictorially illustrate 5 serves a day (Figure 6).

The least popular Facebook posts (ie, seen by ≤30% of participants) were cooking videos with unfamiliar ingredients (eg, squash), meal inspiration posts based on *cliché* ingredients such as avocado, and suggestions to shop at farmer’s markets for cheap vegetables (Figure 6). The uptake of cooking videos and posts made regarding the health benefits of vegetables was moderate (approximately 60%). Overall retention within the social support (Facebook) groups was good. This was measured from when a participant joined the Facebook group until the end of the intervention period regardless of whether the final questionnaire was completed. All but 2 participants were retained.

##### Engagement (Likes)

**Figure 7:**
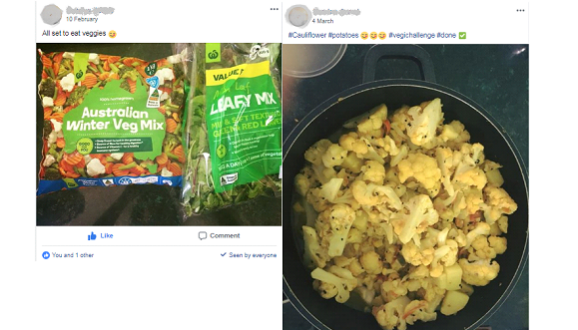
Posts made by members of the Facebook group.

Interaction with posts was limited to likes, with no comments made by participants. Only 1 participant shared their own material within the group as shown in Figure 7. These posts (made by a participant) were very well received with 100% uptake from group participants.

#### Overall Engagement

The data on engagement are displayed in Table 7. No significant differences are detected.

### Program Acceptability

Among participants who completed the follow-up questionnaire, the mean rating given to reflect how much the program was *liked* was 3.3 out of 5 (SD 1.2). On average, the program was rated 3.5 out of 5 (SD 1.2) for how useful it was. The differences between groups were not statistically significant in ratings of *liking* or *usefulness*. However, ratings regarding *ease of use* were more positive for the 2 groups who were allocated the standard self-monitoring app (than people allocated the gaming app for self-monitoring; mean 4.1 [SD 0.85]; *P*=.06).

### Qualitative Analysis

#### App Usability

##### Back-Logging

One of the most commonly cited disadvantages of the app design was the inability to log vegetables eaten from previous days. In total, 8 out of 10 participants interviewed indicated that on several occasions they remembered to log their vegetable intake too late at night (after midnight) or only remembered the following morning, making it difficult to accurately monitor progress. As stated by a female:

I couldn’t go back log in the app and put in what I had forgotten so that made it hard to keep track of when I achieved my goals.

##### Understanding Vegetable Categories

It was frequently reported by participants that they were unfamiliar with the *vegetable categories* at the start of the program and this made it more challenging to quickly navigate through the app to add in vegetables eaten. For some, they stopped logging; however, for others, they found this as a good opportunity to learn the categories that vegetables belonged to. A male stated:

To keep track of the different categories at first was challenging, like which vegetable goes in which category.

##### Offline Functionality

In total, 3 participants noted that the apps’ inability to allow use when offline made it hard to log at the moment of the meal occasion. These participants would revert to logging their meal consumed on the go when they returned to internet connectivity, which often resulted in missed logging opportunities because of forgetfulness. One male summarized this experience with the statement:

The app only loaded when I had internet connection, so when I was out for lunch for example I couldn’t add in what I had eaten and would later forget about it.

##### Self-Monitoring Saturation

Most participants reported that the goal setting and self-monitoring features of the app were the most useful for increasing vegetable intake. As summarized by 1 male:

I found it useful to set a goal of how many veggies to have a day and revise the target over time.

Despite the positive implications of self-monitoring, a majority of those interviewed also indicated that recording with the app discontinued toward the end of the 4-week period and this resulted in a slight drop in their consumption. As stated by 1 female:

It definitely helped to increase vegetable intake at the time when I was using the app, but since I stopped tracking I haven’t been accountable for my intake so I feel I am not as conscious.” It became apparent in the interviews that use of the app for self-monitoring is likely short-lived. Some stated that they would keep the app on their devices for access to recipes and meal ideas after the interventions end.

#### Key Skills Obtained

##### Self-Assessing Adequacy of Intake

Several participants expressed that they learnt to self-assess daily vegetable intake as well as the variety consumed within a couple of weeks of logging. They liked that the app gave them a *benchmark* goal to work against. A few mentioned that they now give consideration to what they eat throughout the day, and if their consumption of vegetables is low, they would compensate through the dinner meal. This is well summarized by 1 male who stated:

I didn’t realize how many serves you are meant to eat and the variety, and now I think about the whole day, like I’ve had some this morning but none all day so I should have some at dinner.

##### Meal Planning and Recipe Modification

Many participants reported that they learnt simple ways to increase their vegetable intake such as adjusting commonly prepared meals so that vegetables featured as an ingredient or including vegetables in meals where they would not usually consume them, such as breakfast. For example, 1 male stated:

I learnt that it was quite easy to increase your vegetable intake without trying too hard or taking too much time, effort or cost. Like just adding some mushrooms or tomatoes to breakfast.

The young adults who were responsible for meal preparation indicated that the simple in-app recipes provided good ideas on how to cook with vegetables on a budget. As summarized by one female:

The recipes were so simple whereas recipes I look up online are not using my usual pantry items. I liked that I could use leftovers in the fridge especially since I am watching my budget a bit more. The pricing was good.

Young adults who did not prepare meals at home indicated that they did not use the recipe function within the app, as stated by one male:

I live with my parents so they do most of the cooking and that’s why I didn’t find the recipes relevant.

#### Motivation Instilled by Facebook Posts

Participants reported that Facebook notifications served as a reminder to log vegetables when they had forgotten to do so. One male stated:

Logging helped me keep track of what I was eating and I did find sometimes the Facebook post would be a reminder to log (especially at the end of the day when I’m busy)*.*” It was indicated that although the tips provided on the Facebook page were motivating, the app was critical for keeping track of progress and maintaining motivation to achieve personal intake goals. As one female stated:

Recipes and tips posted on the Facebook group helped but the app was the most motivating to help me achieve my goals and seeing my progress.

#### Personal Motivations for Participation in the Program

The top 3 reasons for joining the program were: first, being eager to assess whether their current intake was sufficient; second, having the objective of learning ways to add more vegetables to their diet; and finally, a desire to be healthier. As summarized by 1 male participant:

I thought it would be interesting as I have wondered whether I eat enough vegetables. I try to eat healthy as I do a lot of sport and stuff.

## Discussion

### Principal Findings

This feasibility study established that it is technically possible to deliver an app and social media intervention to improve vegetables intake in young adults, although the impact over 4 weeks was negligible and the prevalence of engagement in the target population with a drop off in engagement over time may limit the overall usage and intervention effectiveness. App use halved over the 4-week period. Similar patterns of attrition are observed in other studies delivered using mobile apps [62]. Although game-based incentives (eg, badges) previously have been shown to enhance engagement with digital interventions [63], we did not find any benefit from the addition of incentives. Paradoxically, the group allocated the gamified app had the highest dropout in week 1. Some research in this field has suggested that gamification is not motivating for all users. Points, levels, and leaderboards are usually encouraging for extraverted people, but not necessarily for the population at large [64]. Thus, future work should consider tailoring the use of incentives in a way that is unique to individual personality traits and user preferences. Furthermore, researchers who integrate gaming features into research-based apps should prioritize usability in the design stage, with detailed end-user testing applied. We found that the more complex user experience associated with our gamified app resulted in a lower score from participants for *ease of use*. This may have contributed to the higher drop out among those allocated the gaming app at enrollment, with evidence suggesting that if ease of use of an app is rated low and complexity high, it is possible that participants will disengage [56].

Although reviews of social media–based studies suggest that engagement is likely to decrease over time [29,65], we found that the percentage of Facebook posts viewed was well maintained over the intervention period with a decrease of only 1% between week 1 and week 4. This may be a result of the efforts expended to pretest the Facebook material before use in the intervention, ensuring what was presented was relevant, acceptable, and sustained the interest of the participants. User engagement in the process of development of intervention materials has been recognized by other researchers as a way to improve retention in social media–based studies [65].

Although we attempted to facilitate opportunities for participants to socialize and support each other within the Facebook group, we observed no peer-to-peer interaction and only 1 participant posted their own material within the group. Thus, the social support (Facebook) groups functioned mainly as an information resource and platform to receive support from a dietitian coach. Given that networking platforms such as Facebook are primarily used to maintain existing social relationships rather than foster new connections [66], future research should consider whether using existing connections of participants such as friends and family results in higher peer-based social support and whether this encourages further improvements in behavioral outcomes or engagement with the program.

Unexpectedly, 3 of 4 groups showed a small decline and only the group using the standard goal setting and self-monitoring app had a small increase in vegetable intake. Although these results were not statistically significant, one possible explanation for the absence of notable improvements in intake may be that the dose (period of exposure to the intervention materials) was not long enough to produce behavior change. The positive correlation observed in our study between app engagement (duration) and vegetable intake post intervention supports the upward trajectory that may be observed in vegetable intake if young adults remain engaged over a longer period of time. There is no existing evidence that suggests the optimal amount of time that is required for engagement with a self-monitoring app to yield behavior change. It has been suggested that participants may rely on such apps sporadically, resuming use when they experience difficulty in maintaining the behavior, and thus change could be incremental [67]. A longer term trial, which follows participants for at least 12 months, will be necessary to capture such patterns of engagement and the downstream behavioral outcomes.The observed decrease in vegetable intake in 3 out of 4 groups may be a true effect, a reflection of the fluctuations that are likely occurring in the diets of young adults or may be a result of the reliance on a self-report measure of intake. Evidence exists to confirm selective over-reporting of vegetables in self-report dietary studies [68]. Furthermore, the poor knowledge of standard serving sizes for vegetables at baseline may have caused overestimation of intake, followed by a more accurate report of intake at follow-up after receiving education on vegetable servings through the program. Finally, although the self-report questionnaire was validated, the tool estimated portions using household measures; whereas in the intervention, participants were trained to record intake using the plate method. The impact of this on the reported intake is uncertain. Future research should consider the use of objective measures such as biomarkers on subsamples of the study population to correct for measurement/reporter bias in results.

### Strengths and Limitations

To the best of our knowledge, this was the first study to explore the impact of social support using social media in combination with gaming elements in a nutrition intervention for young adults. A significant strength of this feasibility trial was the development of program components using BCTs, guided by the COM-B framework. A review on the mediators of successful interventions indicated the importance of a systematic approach to selecting BCTs [69]. In addition, all program materials were pretested for acceptability in focus groups with the target audience [46]. This qualitative user-centered approach of addressing the needs of a population is important for the development of tailored interventions for health behavior change [70,71]. Furthermore, measures were taken to streamline the intervention by delivering all material over the Web and over the phone, allowing implementation of the program at scale. The trial also attracted a higher proportion of males than most other nutrition studies [72-74] and participants from a range of socioeconomic status and education levels. Although a wide range of ethnicities were represented among the included participants, the results are not generalizable to all populations, with people of Aboriginal or Torres Strait Islander and South American descent being underrepresented.

One of the main limitations of this research was that it was a feasibility study and not adequately powered for statistical analysis. To measure a change in vegetable intake by one serve, which is considered a clinically significant outcome [75,76], a sample size of 1000 participants would be required in a 4 group factorial study. Given that the intervention period was only 4 weeks, it is also possible that the program was too short to result in behavior change.

### Conclusions

The purpose of this trial was to provide insight into the process of disseminating a social media– and smartphone-based intervention to young adults and assess the acceptability and feasibility of the program. There was no reliance on in-person interaction for dissemination of the program, and the selected platforms (social media, email, and a smartphone app) indicated the feasibility of modern communication technology for the delivery of behavior change interventions to young adults. We found that the uptake of Facebook study materials was better than previously reported in the literature; however, participants engaged passively with no peer-to-peer interaction. Furthermore, engagement with self-monitoring apps decreased over time. We observed that the duration of usage was associated with vegetable intake. Thus, moving forward, this program will require some adaptation and refinement before testing in a larger sample. Further qualitative research with the target audience is needed to develop a deeper understanding of factors influencing engagement, such as personal attributes, and to explore factors that would encourage more effective social support such as using existing social connections or optimizing group size. In addition, consideration should be given to tailoring the use of incentives based on personality traits. Finally, it may be necessary to use a more accurate tool for measuring vegetable intake that does not rely on short self-report questions and instead uses a dietitian-led series of 24-hour dietary recalls or objective measures of change in vegetable intake such as biomarkers.

## Appendix

Multimedia Appendix 1 Educational infographic provided to all participants at baseline via email.

Multimedia Appendix 2 Diagrammatic description of the intervention with protocol flow for each treatment group including randomization, intervention materials, timeline and frequency of measures. a. Standard app has goal setting and self-monitoring feature with recipe database b. Gamified application has goal setting and self-monitoring feature for improving vegetable intake with weekly challenges around eating more vegetables rewarded with badges. Also includes a knowledge quiz on vegetable types and serving sizes as well as a recipe database c. Facebook page provides social support and access to cooking videos, budgeting & meal planning materials, additional educational resources addressing the link between eating more vegetables and health outcomes.

## Abbreviations

BCT: behavior change technique
COM-B: Capability, Opportunity, Motivation and Behavior
SEIFA: Socioeconomic Index For Areas
