# Supplementary figures and images for: Young Adults’ Engagement With a Self-Monitoring App for Vegetable Intake and the Impact of Social Media and Gamification: Feasibility Study
Source: JMIR Form Res. 2019 May 10;3(2):e13324. doi: 10.2196/13324 (PMC6533870; doi:10.2196/13324)

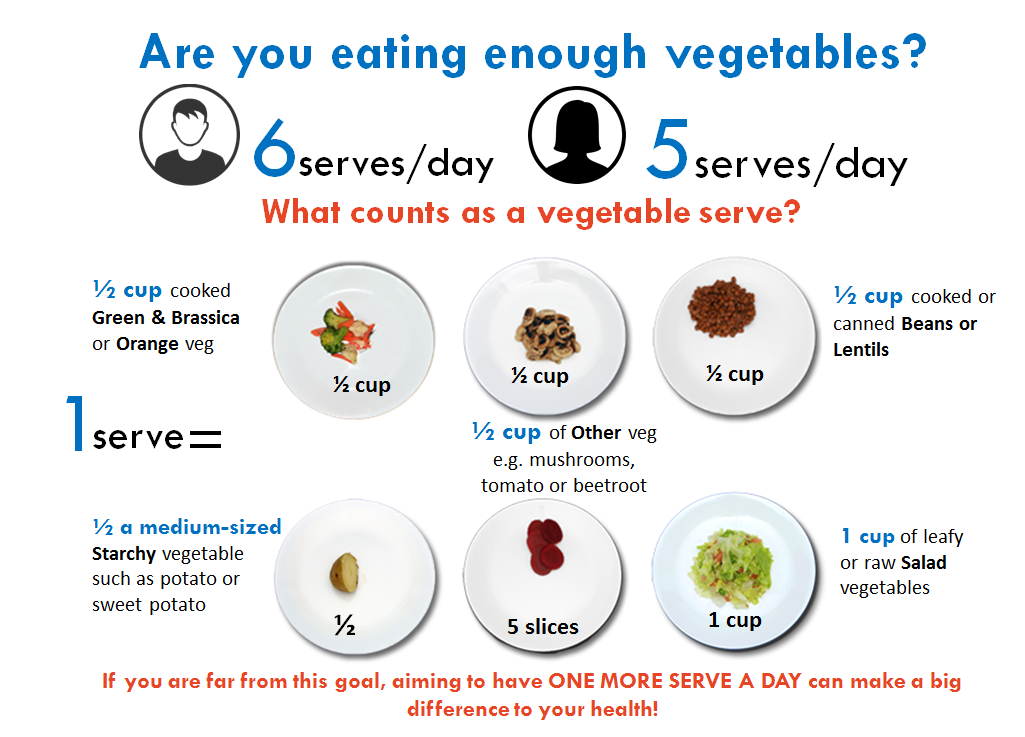

Supplement: Multimedia Appendix 1 [file formative_v3i2e13324_app1.png]
