# Supplementary material for: Young Adults’ Engagement With a Self-Monitoring App for Vegetable Intake and the Impact of Social Media and Gamification: Feasibility Study
Source: JMIR Form Res. 2019 May 10;3(2):e13324. doi: 10.2196/13324 (PMC6533870; doi:10.2196/13324)
Supplement: Multimedia Appendix 2 [file formative_v3i2e13324_app2.docx]

Recruited via flyers, email, news articles, and Facebook advertisements (N=115)
N=115

Completed pre-screening online (N=110)

Eligible Participants (N=97) randomized (2 × 2 factorial design)

| Application type | No Facebook intervention | Facebook intervention |
| --- | --- | --- |
| Standard | n=27 (condition 1) | n=24 (condition 3) |
| Gamified | n=22 (condition 2) | n=24 (condition 4) |

T0 Baseline Assessment: Participants complete the Web-based baseline questionnaire and receive the educational infographic on the recommended serves/day and serving size definitions via email.

**Intervention condition 1 and 2:** Access to **Facebook page** while logging vegetable intake in designated app for 4 weeks

**Intervention condition 3 and 4:** Log vegetable intake in designated app for 4 weeks with no other intervention

Final Assessment (T2) at 4 weeks

Questionnaire re-administered
with the addition of process evaluation questions for engagement

| Completion of T2 Assessment N=47 | | |
| --- | --- | --- |
| Application type | No Facebook intervention | Facebook intervention |
| Standard | n=14 (52%) | n=13 (54%) |
| Gamified | n=9 (41%) | n=11 (46%) |

Random selection of 10 participants to complete 15-min semistructured telephone interview for detailed process evaluation

Follow-up Post-intervention Process Evaluation
